# Antibacterial and cytocompatible silver coating for titanium Boston Keratoprosthesis

**DOI:** 10.3389/fbioe.2024.1421706

**Published:** 2024-09-19

**Authors:** Silvia González Gómez, Maria-Pau Ginebra, Francisco Javier Gil, Rafael I. Barraquer, José María Manero

**Affiliations:** ^1^ Biomaterials, Biomechanics and Tissue Engineering Group, Department of Materials Science and Engineering, Universitat Politècnica de Catalunya. Barcelona Tech (UPC), Barcelona East School of Engineering (EEBE), Barcelona, Spain; ^2^ Barcelona Research Center in Multiscale Science and Engineering, UPC, EEBE, Barcelona, Spain; ^3^ Institute for Bioengineering of Catalonia (IBEC), Barcelona, Spain; ^4^ CIBER de Bioingeniería, Biomateriales y Nanomedicina, Instituto de Salud Carlos III, Madrid, Spain; ^5^ Bioengineering Institute of Technology. Universitat Internacional de Catalunya. Barcelona, Barcelona, Spain; ^6^ Centro de Oftalmología Barraquer, Barcelona, Spain; ^7^ Institut Universitari Barraquer, Universitat Autònoma de Barcelona, Barcelona, Spain; ^8^ Universitat Internacional de Catalunya (UIC), Barcelona, Spain

**Keywords:** Boston keratoprosthesis (BKPro), corneal blindness, titanium (Ti), infection, antibacterial properties, electrodeposition, cytotoxicicity, silver deposition

## Abstract

The Boston Keratoprosthesis (BKPro) serves as a medical solution for restoring vision in complex cases of corneal blindness. Comprising a front plate made of polymethylmethacrylate (PMMA) and a back plate of titanium (Ti), this device utilizes the beneficial biomaterial properties of Ti. While BKPro demonstrates promising retention rates, infection emerges as a significant concern that impacts its long-term efficacy. However, limited research exists on enhancement of BKPros through intrinsic infection-preventing mechanisms. In this regard, metal ions, especially the well-known Ag^+^ ions, are a promising alternative to obtain implants with innate antibacterial properties. However, little information is available about the effects of Ag in corneal tissue, especially within human corneal keratocytes (HCKs). In this work, an electrodeposition treatment using a constant pulse is proposed to attach Ag complexes onto rough Ti surfaces, thus providing antibacterial properties without inducing cytotoxicity. Complete physicochemical characterization and ion release studies were carried out with both control and Ag-treated samples. The possible cytotoxic effects in the short and long term were evaluated *in vitro* with HCKs. Moreover, the antibacterial properties of the silver-treated surfaces were tested against the gram-negative bacterial strain *Pseudomonas aeruginosa* and the gram-positive strain *Staphylococcus epidermidis*, that are common contributors to infections in BKPros. Physicochemical characterization confirmed the presence of silver, predominantly in oxide form, with low release of Ag^+^ ions. Ag-treated surfaces demonstrated no cytotoxicity and promoted long-term proliferation of HCKs. Furthermore, the silver-treated surfaces exhibited a potent antibacterial effect, causing a reduction in bacterial adhesion and evident damage to the bacterial cell walls of *P. aeruginosa* and *S. epidermidis*. The low release of Ag^+^ ions suggested reactive oxygen species (ROS)-mediated oxidative stress imbalance as the bactericidal mechanism of the silver deposits. In conclusion, the proposed electrodeposition technique confers antibacterial protection to the Ti backplate of BKPro, mitigating implant-threatening infections while ensuring non-cytotoxicity within the corneal tissue.

## Graphical Abstract

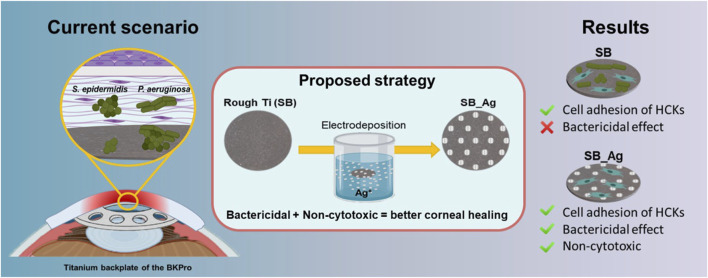

## 1 Introduction

It is estimated that approximately 36 million people worldwide are blind, with corneal disease ranking as the fifth leading cause of blindness, following conditions such as cataracts, glaucoma, and age-related macular degeneration. Over the past few decades, there has been a notable increase in the use of artificial corneas or keratoprostheses (KPros) to restore vision, with the Boston Keratoprosthesis (BKPro) emerging as a prominent choice, boasting over 15,000 implantations worldwide (Nonpassopon et al., 2020). While short-term outcomes of BKPro implantation have been promising, there is a growing concern regarding infections, which can severely impact visual acuity (Avadhanam et al., 2015; Xu et al., 2023).

Bacterial and fungal infections pose significant risks, causing loss of corneal transparency, inflammation, and potential graft melting, ultimately leading to implant failure and loss of vision. Studies have reported high prevalence rates of keratitis and infectious endophthalmitis, reaching up to 17.3% and 12.5%, respectively (Goins et al., 2016; Fry et al., 2018; Prabhasawat et al., 2021). Current treatment approaches primarily rely on pharmacological interventions, which may not always yield successful outcomes. Even with strict adherence to hygiene and medication protocols, established infections are challenging to reverse. Therefore, there is a pressing need to prioritize prevention strategies and, ideally, enhance KPros to incorporate intrinsic infection-preventing mechanisms.

Interestingly, there are limited studies exploring the incorporation of antibacterial properties into BKPro (Gómez et al., 2023). For instance, research utilizing a rabbit keratitis model has suggested the potential of antimicrobial peptides (Tan et al., 2014). However, concerns regarding their stability, high production costs, and susceptibility to proteolytic degradation remain. Another proposed antimicrobial strategy involves the covalent attachment of N, N-hexyl, methyl-polyethylenimine to the BKPro, demonstrating promising inhibitory effects on *Staphylococcus aureus* biofilm formation (Behlau et al., 2011).

Silver is renowned for its antimicrobial properties, though its exact mechanism of action remains incompletely understood. Nonetheless, it has shown efficacy in generating reactive oxygen species (ROS), deactivating enzymes, denaturing DNA, and disrupting bacterial membranes (Li et al., 2010; Mikhailova, 2020).

The use of Ag and its ions to obtain biomaterials with antibacterial properties in the context of increasing antibiotic resistance is of great interest (Godoy-Gallardo et al., 2021). Addressing bacterial adhesion and proliferation at the initial stage is not only essential for preventing immediate infections but also critical for avoiding the formation of biofilms (Bazaka et al., 2011; Campoccia et al., 2013). This is especially important after the implantation of medical devices, as once a biofilm is established, it becomes significantly more challenging to eradicate, often leading to chronic infections and device failure (Costerton et al., 1999; Høiby et al., 2010). Therefore, it is important to develop surface modification treatments with bactericidal effects that are effective during the first hours after implantation. In ophthalmology, silver has been employed in various forms, including artificial eyes, intraocular lenses, topical antibacterial agents, and in the diagnosis and treatment of ophthalmic diseases (Yang et al., 2011; Badur et al., 2017; Ma et al., 2024). Surprisingly, despite ongoing infection challenges, this approach has yet to be explored in keratoprostheses.

In the field of dental applications, our research group has developed a novel electrochemical anodizing process for silver deposition on titanium surfaces, demonstrating significant antibacterial properties *in vitro* without compromising cell viability and with favorable response *in vivo* (Godoy-Gallardo et al., 2015b; 2015a; 2016). The deposited silver particles are robustly anchored, mitigating potential systemic toxicity (Lampé et al., 2019). However, achieving a homogeneous distribution of silver particles with consistent size and shape remains a challenge.

This study aims to utilize the electrochemical anodizing process to create silver particles on rough Titanium BKPro surfaces, ensuring homogeneous distribution, antibacterial efficacy, and compatibility with human corneal keratocytes (HCKs). Optimization of implantation parameters, such as voltage cycles, has been undertaken to enhance efficacy. The physicochemical characterization of modified BKPro surfaces includes scanning electron microscopy (SEM), optical profilometer analysis, contact angle measurements, and X-ray photoelectron spectroscopy (XPS).

For *in vitro* biological characterization, HCKs have been cultured. These primary cells, derived from the human eye, play a crucial role in maintaining corneal stromal integrity. HCKs serve as a valuable *in vitro* model for studying keratocyte differentiation, corneal injury, and related diseases. While previous studies have utilized human foreskin fibroblasts (HFFs), there is not any information about the effect of silver on HCK cells (Godoy-Gallardo et al., 2015a; 2015b). Finally, the antibacterial efficacy was assessed against *Pseudomonas aeruginosa* and *Staphylococcus epidermidis*, both significant contributors to ophthalmic infections (Robert et al., 2012; Wagoner et al., 2016a).

In summary, the increasing use of keratoprostheses highlights the critical need for effective infection prevention strategies. While various antimicrobial strategies have been explored, including antimicrobial peptides and covalent attachment of antibacterial agents, the potential of silver remains unexplored in the context of keratoprostheses.

This study aims to develop a novel electrochemical anodizing process for silver deposition on rough Ti. By optimizing implantation parameters, we aim to achieve robust antibacterial efficacy against common pathogens in ophthalmic infections while maintaining compatibility with human corneal keratocytes. This research represents a significant step forward in addressing the challenge of infections associated with keratoprostheses, with potential benefits for patient outcomes and healthcare burdens.

## 2 Materials and methods

### 2.1 Sample preparation and cleaning

Commercially pure titanium grade 2 rods were machined to produce disks (10 mm diameter, 2 mm thickness). The disks were sandblasted to achieve rough surfaces such as those presented by commercial BKPro (Ra 1.9 ± 0.3 μm) using alumina (Al_2_O_3_) particles of p80 size. Afterwards, the disks were ultrasonically cleaned with distilled water, ethanol, and acetone (3 × 5 min each), and stored dried.

### 2.2 Electrodeposition treatment

The electrochemical anodization technique was used for the deposition of silver on the surface of the samples. The process has been described elsewhere (Godoy-Gallardo et al., 2015a), however, in brief, samples were immersed in an electrochemical cell using a 0.1: 0.2 M (AgNO_3_: Na_2_S_2_O_3_) solution as electrolyte. The Ti disks were used as working electrode and a platinum sheet was used as counter electrode. The anodizing process was controlled with a Potentiostat (PARSTAT 2273, US). All reactions were carried out under constant stirring and at room temperature.

An ascendant rectangular pulse was reported previously, which increased by 10 mV each step until it reached a final voltage of 10 V (Initial potential (E_i_) = 0 V, Final potential (E_f_) = 5 V, Step time (S_T_) = 500 m, Step Height (S_H_) = 10 mV, Pulses width (P_W_) = 100 m). An entire cycle with this type of pulse lasted 250 s (Godoy-Gallardo et al., 2015a).

**FIGURE 1:**
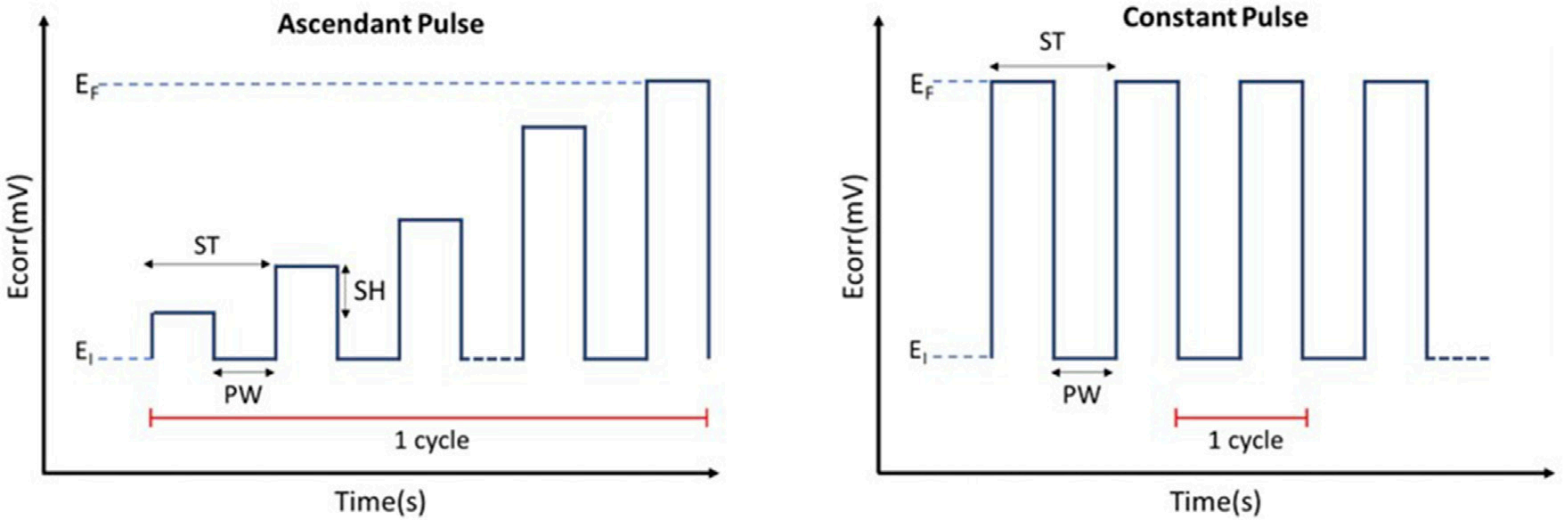
Comparative diagram of the ascendant pulse used previously (Godoy-Gallardo et al., 2015a) and the constant pulse tested in this work. SH= Step Height, PW= Pulse Width, ST = Step Time.

To improve the homogeneity of the silver deposition and shorten the process time, some modifications of the electric pulse were introduced. Thus, instead of using a staggered ascending pulse, a sequence of rectangular pulses ranging from 0 V to 10 V was used directly. Since the increase in voltage occurs directly, this pulse significantly shortens the process time. The diagram of both types of pulses is shown in Figure 1. After the anodization treatments the samples were sonicated in ethanol, water, and acetone for 15 min each. Rough Ti samples (SB) were used as control and the Ag-treated samples were mentioned as SB_Ag.

### 2.3 Physicochemical characterization

#### 2.3.1 Morphological analysis

Surface topography of the samples was studied by means of scanning electron microscopy (SEM) (PhenomWorld ProX, Netherlands). Electronic images were taken at a working distance of 4 mm and a potential of 10 kV using backscattered electrons (BSE). Three samples of each condition were observed.

#### 2.3.2 Roughness analysis

Surface roughness was assessed using an optical profilometer (Micromesure 2, STIL, France). A representative map scanning of the surface was acquired using three samples of each condition. The measurements were analysed using the MountainsMap^®^ Topography (Digital Surf, France).

#### 2.3.3 Contact angle analysis

The wettability of the samples was determined by the sessile drop method using a contact angle system OCA15 plus (Dataphysics, Germany).

The measurements were done at room temperature (25°C) using ultrapure water and diiodomethane as wetting liquids (drop volume of 1 μL). The measurements were performed per triplicate in each sample using three samples per condition. The water-contact angles below 90° were considered hydrophilic and above 90° were considered hydrophobic (Law, 2014).

The Wenzel approach was used to calculate the angles formed between the water drop and the surface with SCA 20 software (Dataphysics, Germany). The Wenzel approach assumes that the water drop can penetrate the surface texture of the solid (Kim et al., 2016).

Therefore, for rough samples, it is necessary to calculate the real contact area between solid and liquid also called roughness ratio factor (r). This parameter is related with Sdr as defines the equation below (Equation 1) (Chi et al., 2016):

$$r=1+\frac{Sdr}{100}$$ (1)

The roughness ratio equals one for ideal flat surfaces and is higher than one for rough surfaces. Adding the effect of the roughness in the young’s equation is obtained the Wenzel’s equation (Equation 2 (Wenzel, 1949).).

$${\mathrm{Cos}\theta }_{m\;}={r\cdot \mathrm{Cos}\theta }_{m\;}$$ (2)

In the Wenzel’s approach, θ_m_ is the apparent angle that is measured and θ_γ_ corresponds to the Young’s angle that is corrected with the roughness ratio (Belaud et al., 2015; Czifra and Barányi, 2020) .

The surface free energy (SFE) and its dispersive and polar components were calculated using the Young–Laplace (Equation 3) and the Owen-Wendt (Equation 4) equations (Della Volpe et al., 2004; Zhang, 2020).

$$\gamma_{S}=\gamma_{\mathrm{SL}}+\gamma_{L}\cdot \cos\theta$$ (3)

$$\gamma_{L}\left(1+\cos\theta \right)=2({\left(\;\gamma_{L\;\cdot }^{d}\gamma_{S}^{d}\right)}^{1/2}+{\left(\;\gamma_{L\;\cdot }^{p}\gamma_{S}^{p}\right)}^{1/2}$$ (4)

#### 2.3.4 Surface chemical characterization

The surface composition of the samples was analyzed by X-ray photoelectron spectroscopy (XPS) with an Mg anode XR50 source operating at 150 W and a Phoibos 150 MCD-9 detector (D8 advance, SPECS Surface Nano Analysis GmbH, Germany). Spectra were recorded with pass energy of 25 eV at 0.1 eV steps at a pressure below 7.5·10^−9^ mbar. The peak fitting and the spectral analysis were performed using Casa XPS software (Version 2.3.16, Casa Software Ltd., United Kingdom) and all binding energies were referenced to the C1s signal (284.8 eV). Two samples of each condition were studied. The ratios silver/titanium and sulfur/titanium were calculated from the measured elemental surface composition.

#### 2.3.5 Ion release

The Ti and Ag ions released from the samples were evaluated according to the ISO-10993–12 standard using an Inductively Coupled Plasma Mass Spectrometry (ICP-MS) equipment (ICP-MS, Agilent 5100 SVDV ICP-OES, CA, US) for the analyses (International Organization for Standardization, 2012). Each sample was immersed in 3.5 mL of Hanks’ solution (1 mL of Hanks’ solution per Gram of porous material) and incubated at 37.5°C for 1 h, 1 d, 3 d, 7 d and 14 d as previously described (Rodríguez-Contreras et al., 2020).

One mL of the incubated Hanks’ solutions was taken, filtered, and diluted 1:2 in 2% nitric solution an analyzed through ICP-MS to obtain the ion release values. The same volume taken was replaced with Hanks’ solution to maintain the initial volume. The tests were performed in triplicate using three samples of each condition. All measurements obtained are expressed in ppb (parts per billion). The lower detection limits of the ICP-MS equipment used were 0.10 ppb for Ti and 0.83 ppb for Ag detection.

### 2.4 Biological characterization

#### 2.4.1 Cell assays

##### 2.4.1.1 Cell culture

Human Corneal Keratocytes (HCK) (Cell applications Inc., US) were cultured in Human Corneal Keratocyte Media (Cell applications Inc., US) at 37°C in a humidified incubator and 5% (v/v) CO_2_, changing the medium every 2 days. Confluent HCKs were detached from the culture flask by incubation with TrypLE (Invitrogen, US) for 5 min. All experiments were performed at passage 3-4.

##### 2.4.1.2 Cell adhesion and morphology

To study the effect of silver in the adhesion and the morphology of HCKs, at least 10,000 cells were seeded on all samples of each experimental condition and incubated in HCK medium for 6 h at 37°C to enable accurate comparison between the two groups. After 6 h of incubation, cells attached to the surfaces were fixed for 30 min with 4% (w/v) paraformaldehyde (PFA). Cell adhesion and morphology were assessed through nuclei and actin fiber staining in the control and functionalized samples. To this end, cells were permeabilized with 500 μL/disk of 0.05% (w/v) Triton X-100 in phosphate-buffered saline (PBS) for 20 min and blocked with 1% bovine serum albumin (BSA) (w/v) in PBS for 30 min. Washings between steps were all performed with PBS-Gly (PBS containing 20 mM of glycine) for 3 × 5 min. Then, 100 μL/disk of primary antibody mouse anti-vinculin (1:100 in 1% (w/v) BSA in PBS) were incubated onto the Ti samples for 1 h.

The secondary antibody, anti-mouse Alexa 488 (1:2000), and phalloidin-rhodamine (1:300) were both incubated in triton 0.05% (w/v) in PBS for 1 h in the dark. In a final step, nuclei of cells were also stained using 500 μL/disk of 4′,6-diamidino-2-phenylindole (DAPI) (1:1000) in PBS-Gly for 2 min in the dark. Titanium disks were then mounted on microscope slides and analyzed by fluorescence microscopy (Nikon E600, Tokyo, Japan). The number and spreading of cells attached on each surface was assessed using ImageJ 1.46R software (NIH, US). Cell numbers were calculated by counting cell nuclei on five fields per disk and measuring the mean values. The area of adherent cells was measured for at least 10 cells for each sample and averaged for three samples for each condition. The shape descriptors (circularity and Aspect Ratio (AP)) were calculated using the ‘Shape Descriptors’ plugin in ImageJ. All cellular studies were performed per triplicate using three samples per condition.

##### 2.4.1.3 Cytotoxicity assays

The potential cytotoxic effect of the samples was evaluated according to the ISO 10993–5 and ISO 10993–12 standards using HCKs (International Organization for Standardization, 2009; International Organization for Standardization, 2012). The samples were sterilized by immersion in ethanol 70% during 30 min. After sterilization, the samples were rinsed thrice with sterile PBS to remove any residual ethanol. Next, the samples were placed in the wells of a 48-well plate, and 500 µL of Corneal Keratocyte Media (Cell applications Inc., US) was added to each well. The samples were incubated with medium for 72 h to allow any potentially harmful substances to be released from the samples into the medium. The medium incubated with the samples, referred to as the extract, was then used to prepare 1:1, 1:10, 1:100, and 1:1000 dilutions. These studies were performed per triplicate using three samples of each condition to prepare the extracts each time.

Thereafter, 5000 cells per well were seeded into the wells of a 96-well tissue culture polystyrene plate and incubated with media for 24 h. Then, the culture medium was replaced with the extract dilutions, and the cells were cultured for an additional 24 h. At the end of this incubation period, the medium was aspirated, and the wells were rinsed thrice with PBS to remove residual culture media. To determine the number of adherent cells, after removing the supernatant, the cells were incubated with 10% (v/v) PrestoBlue™ (Invitrogen, US). Following the incubation period, the supernatants were collected, and their fluorescence measured using a Synergy HTX multimode reader (Agilent, CA, US).

Finally, to convert the fluorescence read-out into cell numbers, a standard curve was used. The standard curve was obtained by plotting the fluorescence values obtained regarding a known number of cells. The cytotoxicity results were normalized to the SB condition.

The cell viability was calculated following Equation 5, were Fluo_sample_ is the fluorescence of the supernatant of the cells incubated with the different extracts of one sample type (SB samples or SB_Ag samples), Fluo_C+_ is the fluorescence of the supernatant of the cells incubated with culture media and Fluo_C-_ is the fluorescence of the culture medium without cells.

$$Cell\;viability\;\%=\frac{{Fluo}_{sample}-\;{Fluo}_{C-}}{{Fluo}_{C+}-\;{Fluo}_{C-}}*100$$ (5)

As stated in the ISO standard, a material is not considered cytotoxic if viability value (%) greater than 80% is obtained using the previously mentioned formula (International Organization for Standardization, 2009).

##### 2.4.1.4 Cell proliferation

To assay the proliferation of HCKs, at least 10,000 cells were seeded on each sample and incubated with Human Corneal Keratocyte Media. Every 2 days, medium was exchanged. At the timepoints of 1, 3, 7, and 14 days, the medium was aspirated, the samples were washed with PBS and incubated with Presto Blue (PB) (Invitrogen, US). The supernatants were collected, and their fluorescence at 590 nm was measured. The fluorescence was interpolated in the calibration curve as previously described to know the cell number. Proliferation studies were performed per triplicate using three samples per condition.

#### 2.4.2 Bacterial assays

##### 2.4.2.1 Bacterial culture

Bacterial assays were performed with gram-negative strain *P. aeruginosa* (CECT 110*,* from the Colección Española de Cultivos Tipo, Valencia, Spain) and gram-positive strain *S. epidermidis* (CECT 231, from the Colección Española de Cultivos Tipo, Valencia, Spain), as both are common pathogens found in ophthalmic infections (Robert et al., 2013; Behlau et al., 2014).

*Pseudomonas aeruginosa* was growth in Luria broth (Scharlab SL, Spain) and *S. epidermidis* was growth in Brain heart infusion broth (Scharlab SL, Spain). Cultures were incubated overnight at 37°C before each assay.

##### 2.4.2.2 Bacterial reduction and dead

Samples were sterilized with 70% ethanol for 5 min and then washed twice with PBS to remove any remaining ethanol. Next, a bacterial suspension was prepared adjusting the optical density of each bacterial suspension to 0.2 ± 0.01 absorbance units (AU) at 600 nm, giving approximately 1·10^8^ colony-forming units (CFU) ml^-1^ for each strain. Subsequently, 1 mL of the previously adjusted bacterial solution was added onto the sterilized samples. The samples were then incubated with the bacteria for 4 h at 37°C under static conditions. This timepoint was selected to assess the bactericidal effect of Ag particles in the early hours, providing insights into initial adhesion dynamics before significant biofilm formation occurs (Thi et al., 2020). Afterward, the supernatant was removed, and the samples were washed with PBS to remove non-adhered bacteria. The samples were then stained with a solution on PBS of the LIVE/DEAD BackLight Bacterial Viability Kit (ThermoFisher, US) at room temperature in the dark for 15 min. Bacterial studies were performed in triplicate, using three samples per condition. A Zeiss LSM 800 confocal microscope (Carl Zeiss, Germany) equipped with a 63x lens was used to observe the samples. At least five images were taken randomly across each sample surface. The images of the SYTO-9 and Propidium iodide (PI) staining were acquired using Zen 2.3 software (Carl Zeiss).

The bacterial reduction (%) was calculated using Equation 6, where A is the total amount of bacteria in the SB samples and B is the total amount of bacteria in the SB_Ag samples (Mascarenhas et al., 2022).

$$Bacterial\;reduction\;\left(\%\right)=\frac{A-\;B}{A}*100$$ (6)

The percentage of bacterial dead was calculated by dividing the bacteria stained with PI (red bacteria) by the total bacteria (sum of bacteria stained with SYTO-9 and bacteria stained with PI) (Equation 7).

$$Bacterial\;dead\;\left(\%\right)=\frac{\text{red}\;\mathrm{bacteria}}{\text{total}\;\mathrm{number}\;\mathrm{of}\;\mathrm{bacteria}}*100$$ (7)

##### 2.4.2.3 Bacterial adhesion and morphology by scanning electron microscopy

After bacterial damage assays the samples were prepared for SEM observation. Thus, samples were dehydrated with grading of ethanol (30%, 50%, 70%, 90%, 95% and 100%) of 15 min in each step. Next, samples were covered with charcoal and observed with SEM. Electronic images were taken at a working distance of 4 mm and a potential of 10 kV using secondary electrons. Three samples of each condition were observed.

### 2.5 Statistical analysis

The data presented in this study is given as mean values of each condition ± standard deviations. Statistically significant differences between groups were assessed by Minitab 19 software (Minitab, EE. UU). The data of the samples were previously tested for normality. Afterwards, t-student test or Man-Whitney test were carried out to obtain the *p*-value depending on if the data followed or not a normal distribution. The confidence interval was set as 95% unless otherwise specified.

## 3 Results

### 3.1 Physicochemical characterization

#### 3.1.1 Surface topography

**FIGURE 2:**
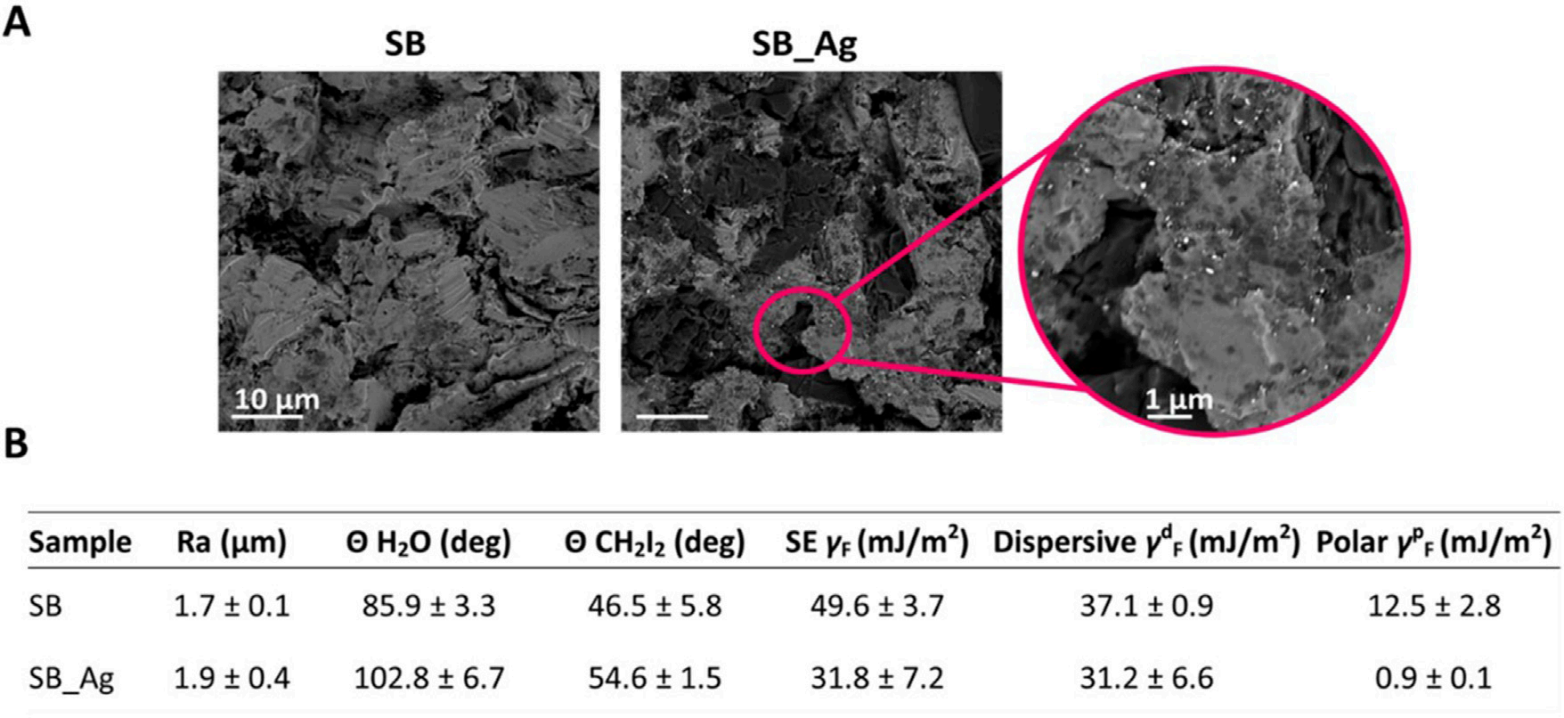
**(A)** Scanning electron microscope (SEM) images of the Ti rough samples (SB) and rough samples with silver (SB_Ag), where the white particles are the silver deposits. Scale bars equal 10 μm and 1 µm. **(B)** Results of the measurements of Average Roughness (Ra), contact angle measurements (θ) with water and diiodomethane, Surface Energy (SE), and the SE components (dispersive and polar). All the values represent mean ± standard deviation (SD) in SB and SB_Ag samples.

The surface of samples was analyzed by SEM as shown in Figure 2A. BSE-SEM images showed a matrix of Ti with dark alumina particles from the sandblasting process, in both experimental groups. In the SB_Ag samples the silver particles were appreciated when increasing the magnification. Silver deposits were homogeneously distributed over the entire surface. The electrodeposition process did not change the texture of the surface.

#### 3.1.2 Wettability and SFE

Next, the roughness and wettability of the SB and SB_Ag samples were studied (Figure 2B). The values of roughness measurements were very similar for both experimental conditions. As it had been observed by SEM, the electrodeposition process does not produce significant changes in the topography of the samples.

The water contact angle values were higher for SB_Ag samples compared to SB. This change in the wettability of the samples indirectly indicates the presence of the silver deposits (Figure 2B).

The contact angle values for diiodomethane were higher after the electrodeposition process. Regarding the components of SFE, the values of the dispersive component were similar for SB and SB_Ag samples, while the value of the polar component decreased in the SB_Ag samples. Therefore, the SB_Ag samples exhibited hydrophobic behavior, with lower SFE values primarily attributed to the decrease in the polar component.

#### 3.1.3 XPS studies

The successful immobilization of the silver particles into the Ti surfaces was further corroborated by XPS. With this aim, the chemical composition of the SB and the SB_Ag samples was studied.

**FIGURE 3:**
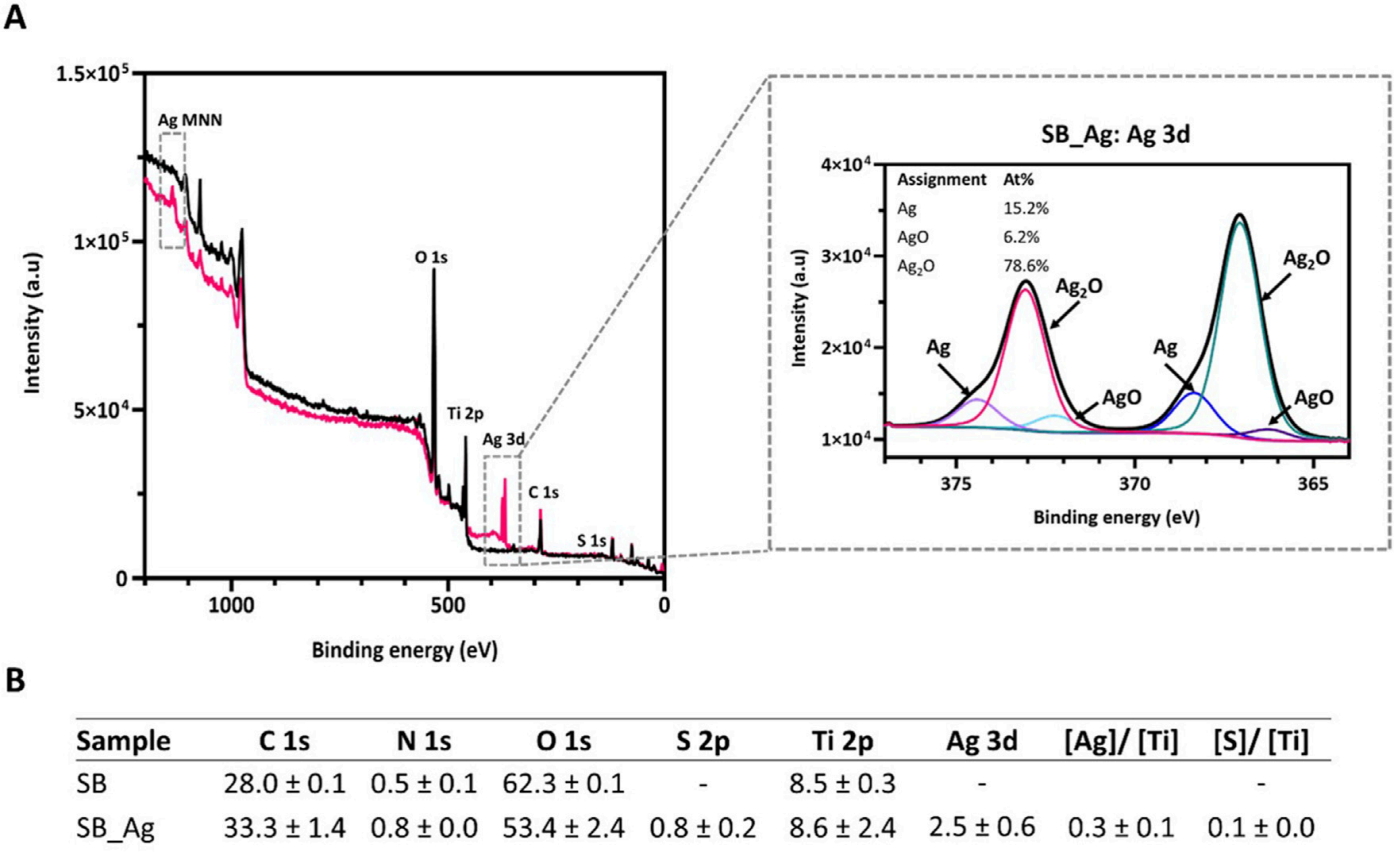
**(A)** X-ray photoelectron spectroscopy (XPS) survey spectra of SB (black) and SB_Ag samples (pink). Decomposed high-resolution Ag3d core level spectrum of SB_Ag samples. **(B)** Chemical composition (atomic percentage) measured by XPS (mean ± standard deviation). (SB: sandblasted Ti; SB_Ag sandblasted Ti with silver).

The characteristic signals corresponding to C 1s, O 1s, N 1s, S 2p, Ag 3d, and Ti 2p are shown in Figure 3B. The high-resolution spectra of Ag3d was also acquired (Figure 3A).

After the electrodeposition treatment there was an increase in the C 1s signal whereas the O 1s and the Ti 2p decreased. The Ag3d and S2p signals were only detected in the SB_Ag samples. The presence of carbon was attributed to residual or reabsorbed atmospheric organic contamination in both sample types (Paredes et al., 2017). A small amount of N contamination was also observed. The increase in carbon and nitrogen after the electrodeposition treatment was associated with the increased handling of the samples.

The presence of silver in the treated surfaces was corroborated by the appearance of the characteristic double peak in the Ag 3d region, in addition to the appearance of the characteristic signal in the Ag MNN region (Figure 3A). The S/Ti and the Ag/Ti ratio obtained were according to similar studies (Godoy-Gallardo et al., 2015a) (Figure 3B).

The high-resolution spectrum of Ag3d revealed the presence of two peaks separated by 6eV: Ag3d_5/2_ and Ag3d_3/2_. The Ag3d_5/2_ peak could be divided into three peaks related to metallic Ag (368.4 eV), Ag_2_O (367.1 eV) and AgO (366.2 eV). The Ag3d_3/2_ peak was formed also by metallic Ag and the Ag oxides. The ratio Ag/Ag oxides was 0.18 indicating prevalence of the oxide forms.

**FIGURE 4:**
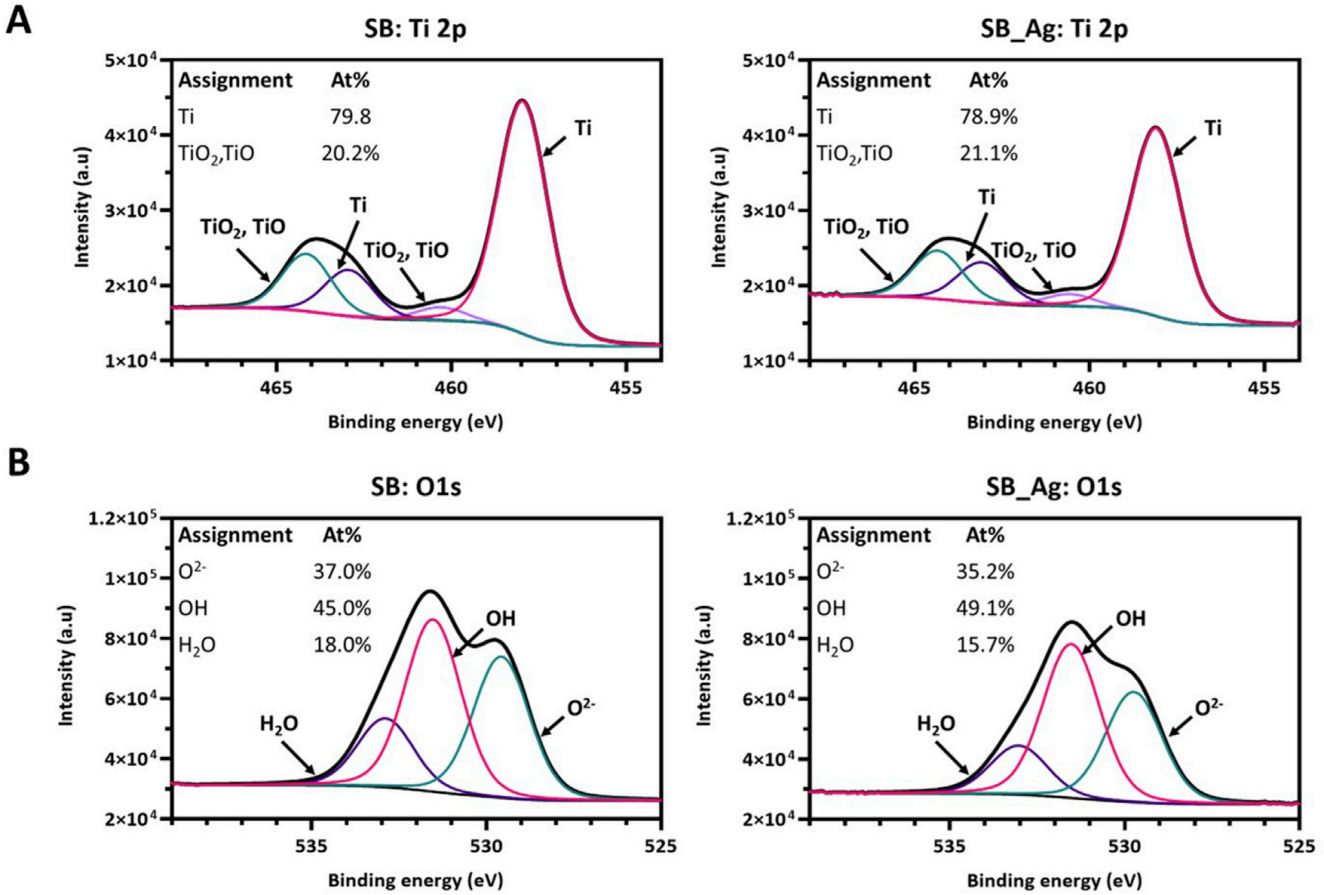
**(A)** Decomposed high-resolution Ti2p core level spectrum of SB and SB_Ag samples. **(B)** Decomposed high-resolution O1s core level spectrum of SB and SB_Ag samples (SB: sandblasted Ti; SB_Ag sandblasted Ti with silver).

To further investigate the changes in the surface composition after the electrodeposition treatment, the high-resolution spectrums of O1s and Ti2p were also acquired (Figure 4).

The Ti2p curve with the electron level Ti 2p3/2 at 458 eV and the Ti2p1/2 at 464 eV, were deconvoluted in three peaks originating from the metallic (Ti) and oxide states (TiO2 and TiO) in SB and SB_Ag samples. The peaks were separated by 6.16 eV (Figure 4A). The ratio Ti/Ti oxides was 3.95 for SB samples and 3.73 for SB_Ag, indicating prevalence of the Ti metallic form towards the Ti oxides.

In the high-resolution study of the O 1s spectrum were identified three contributions: (O_1_) at 529.6 eV attributed to Ti oxides (O^2−^), (O_2_) at 531.5 eV attributed to hydroxyl groups (−OH) and (O_3_) at 532.8 eV associated to H_2_O (Figure 4B). In the SB_Ag samples there was a decrease in the O^2−^ and the H_2_O when comparing with the SB samples although this decrease was not significant.

#### 3.1.4 Ion release

**FIGURE 5:**
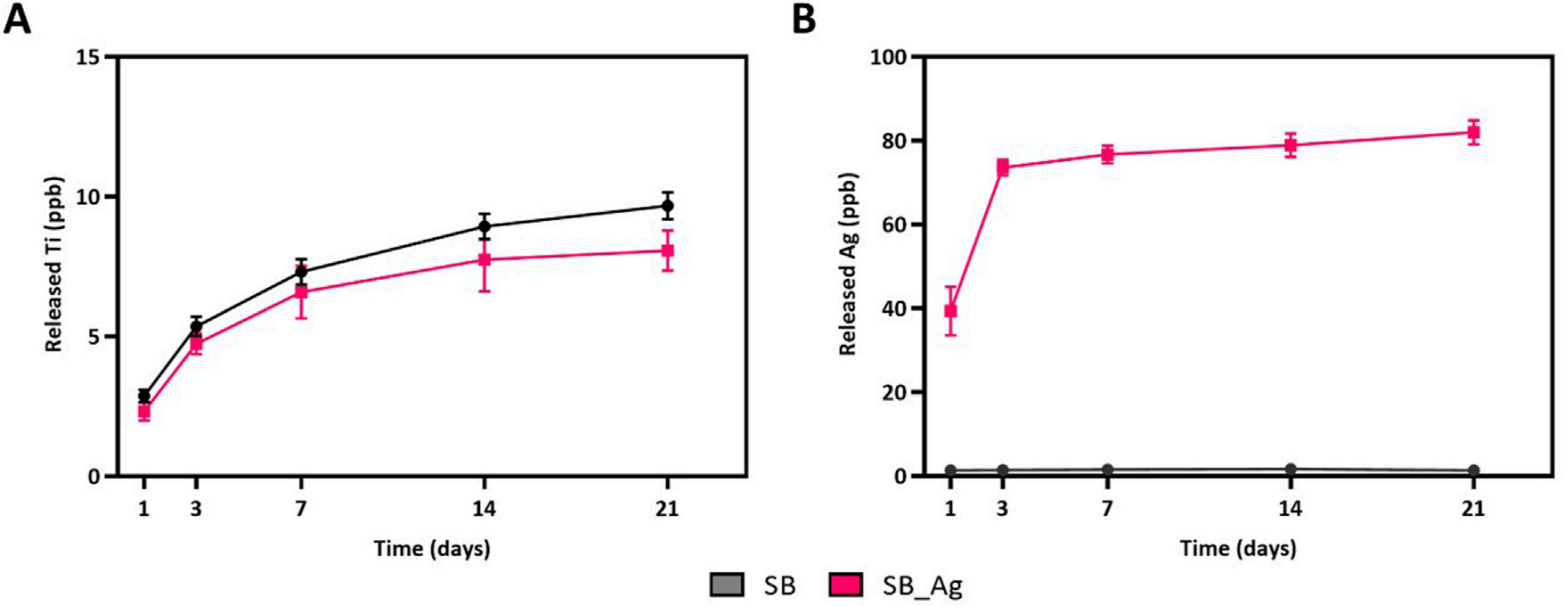
**(A)** Ti released ions in parts per billion (ppb). **(B)** Ag released ions in parts per billion (ppb) from the rough Ti (SB) and the rough Ti with Ag particles (SB_Ag) at different time points.

To determine the release of Ag and Ti ions, an ion release study was performed following the ISO-10993–12 as shown in Figure 5.

The Ti and Ag release curves showed an initial fast release followed by stabilization after 3 days of incubation. The amount of Ti ions released was very low and slightly higher for the SB samples. For Ag, the release was only detected in the SB_Ag samples. Ag^+^ ions were detected after 24 h at a concentration of 39.32 ppb. After 72 h, the released amount was 73 ppb. The release level was maintained for 21 days, with a final concentration of 85 ppb.

### 3.2 Biological characterization

#### 3.2.1 Cell adhesion and morphology

To detect possible early adverse effects of the Ag particles, cell adhesion tests were performed.

**FIGURE 6:**
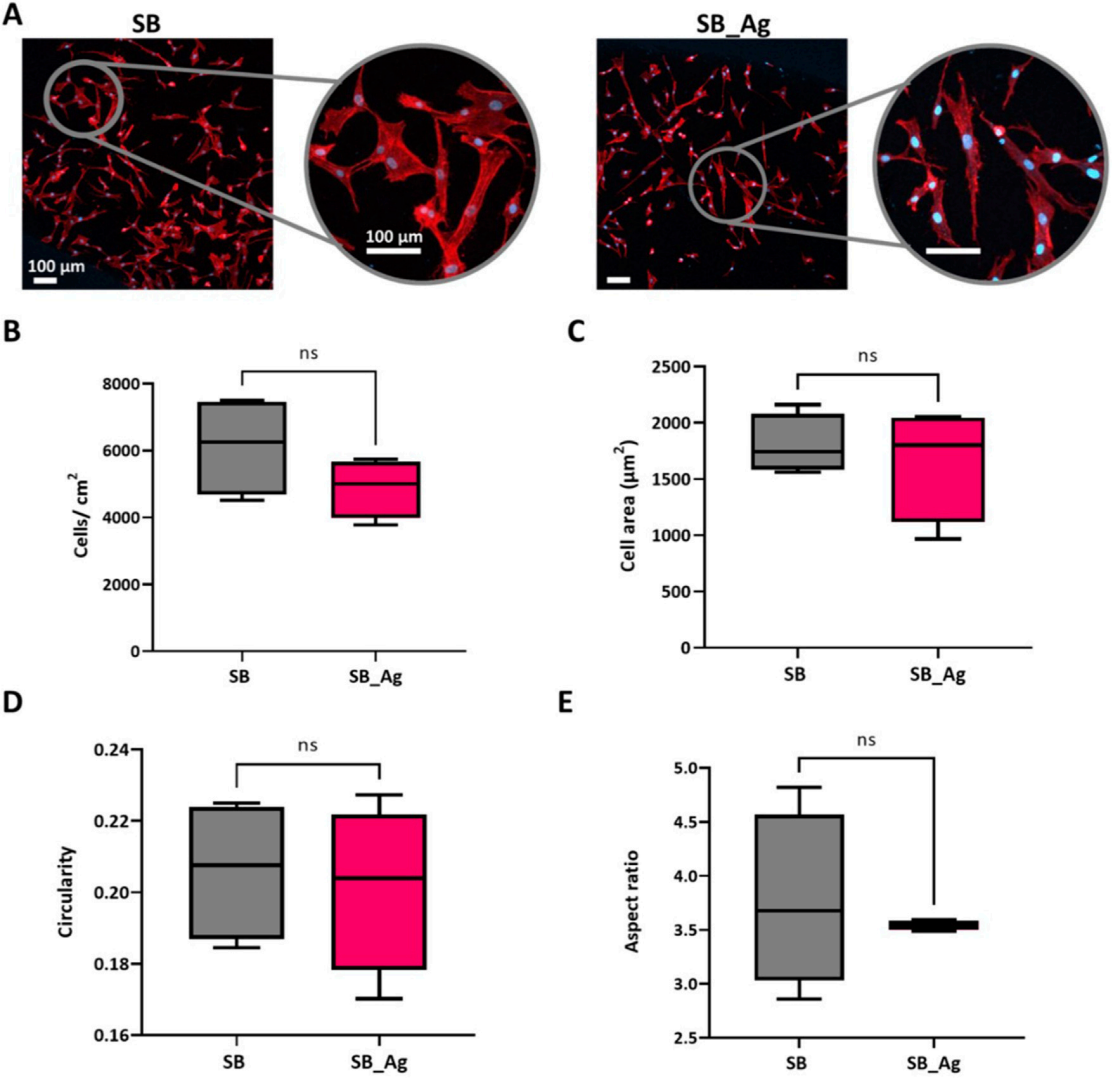
Human corneal keratocytes (HCKs) behaviour on Ti surfaces after 6 h of culture. SB: sandblasted Ti, SB_Ag: sandblasted Ti with silver electrodeposited particles. **(A)** F-Actin and nucleus immunostaining (scale bar = 100 μm). **(B)** HCKs adhesion **(C)** Cell area **(D)** Cell circularity **(E)** Cell aspect ratio. Each condition was replicated in triplets (n = 3) and five pictures per sample were used to identify cell adhesion and calculate shape descriptors. Abbreviations: “ns” indicates not statically significant (*p* > 0.05)

The tests showed a decrease in the number of attached HCKs on the Ag samples, although this decrease was not statistically significant (Figure 6B). In both conditions, HCKs were observed to be distributed over the entire surface and were well spread, except for a few isolated cells that exhibited a more rounded morphology (Figure 6A). The cell spreading was similar in both cases showing no significant differences between both groups (Figure 6C), indicating greater variability in the Ag samples.

Circularity is a shape descriptor that indicates how closely the cells resemble a perfect circle. Initially, cells typically have a more circular shape, but over time, they adapt and take on a more spread shape. In the present study, very similar circularity values close to 0 were obtained for both SB and SB_Ag, indicating that the HCKs do not present a rounded shape. This observation was also confirmed by the immunofluorescence images. These findings are a positive sign, indicating that the cells can properly adhere to the sandblasted topography as well as to the same topography with silver agglomerates. Thus, the presence of Ag does not affect the proper adhesion of cells to the surface as observed in the fluorescence images (Figure 6D).

Regarding aspect ratio, it was observed that HCKs from SB_Ag samples exhibited a more elongated and narrower phenotype compared to control samples. This resulted in lower aspect ratio (AP) values, as depicted in Figure 6E. In contrast, the presence of more HCKs spread and with higher area in the SB samples, resulted in a larger deviation in the AP values. Recent studies have shown that keratocytes are susceptible to physical reprogramming influenced by factors such as the hydrophobicity of the substrate on which they are cultured (Li et al., 2021). Consequently, we believe that the observed changes in cell shape and behavior may be due to the differences in surface composition and wettability, which could influence cell morphology and lead to the more elongated and narrower phenotype observed in HCKs from SB_Ag samples.

#### 3.2.2 Cytotoxicity and proliferation studies

**FIGURE 7:**
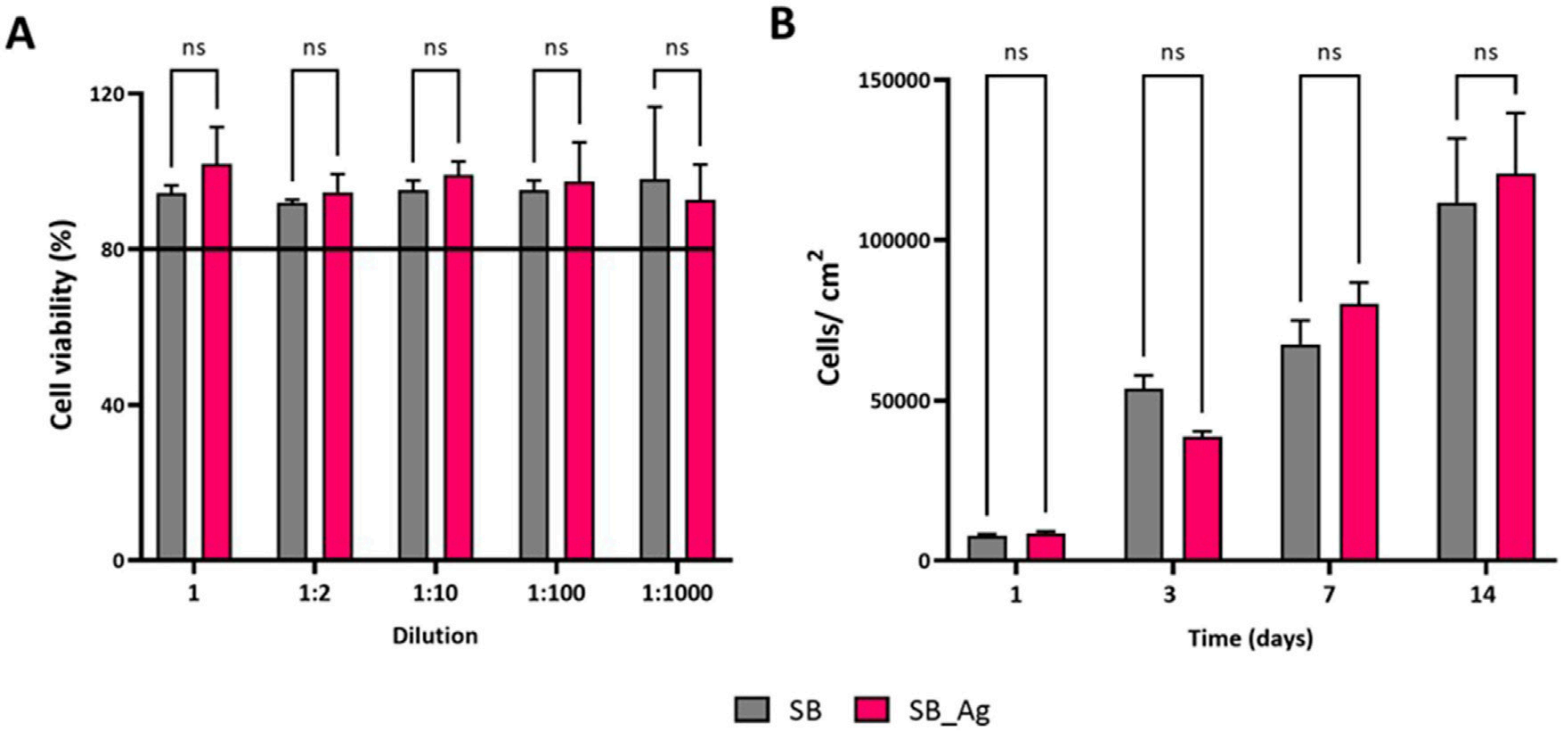
**(A)** Cell viability of Human corneal keratocytes (HCKs) **(B)** Cell proliferation after 1,3, 7 and 14 days of culture. Quantification of cells was done with Presto Blue assay. SB: sandblasted Ti, SB_Ag: sandblasted Ti with silver electrodeposited particles. Abbreviations: “ns” indicates not statically significant (*p* > 0.05).

The cytotoxic potential of the Ag particles was evaluated according to ISO 10993–5 (Figure 7A). Viability did not decrease for any dilution when tested with HCKs. Samples from both groups exhibited viability values above 80%, indicating the absence of harmful compound release from the Ag particles even with the undiluted extract, which had an estimated content of 73 ppb of Ag^+^.

The next step was to check the effect produced by the presence of silver particles on cell proliferation. As explained in the experimental methods section, HCKs were incubated with the functionalized surfaces and the controls for 1, 3, 7 and 14 days (Figure 7B). The first day, a similar number of cells were counted in both conditions, on day 3 there were more cells in SB condition, on day 7 slightly more cells were detected in the SB_Ag samples and on day 14 the number of cells was very similar in both groups. The differences observed were not statistically significant. The absence of differences in the cytotoxicity and proliferation assays with the control samples underscores the lack of negative effects observed after long exposure periods to the Ag agglomerates.

#### 3.2.3 Antibacterial studies

**FIGURE 8:**
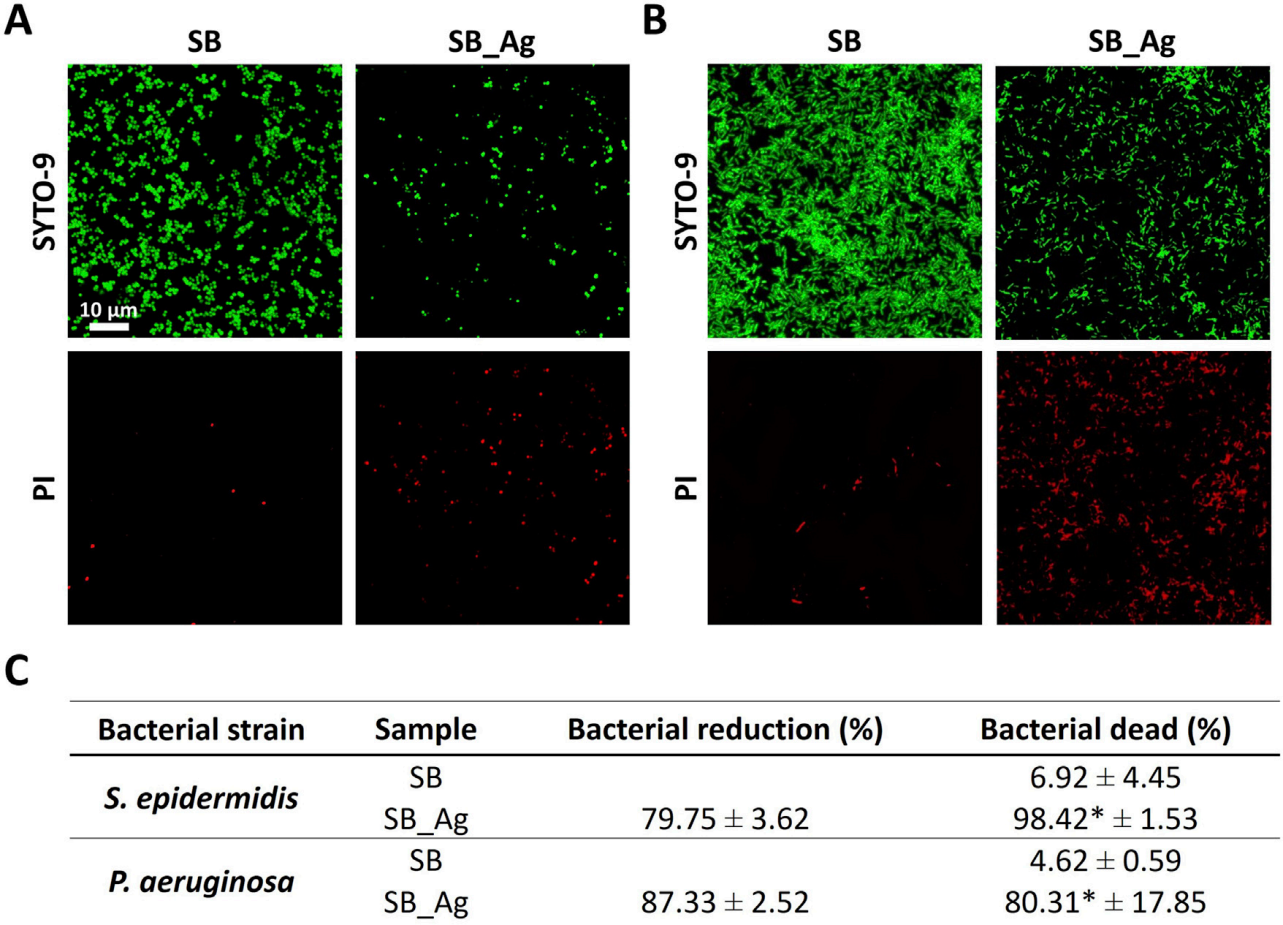
Live/dead staining to assess bacterial damage **(A)** 4 h adhesion of Staphylococcus epidermidis **(B)** 4 h adhesion of Pseudomonas aeruginosa. **(C)** Percentage of bacterial adhesion compared to SB control samples and bacterial dead percentage (mean ± standard deviation). SB: rough Ti, SB_Ag: rough Ti with silver electrodeposited particles * Indicates statistically significant differences with the control condition (*p* < 0.05).

After checking the non-cytotoxicity of the silver samples, the antibacterial effect of the silver particles was tested. For this purpose, bacterial adhesion assays were performed at 4 h with *P. aeruginosa* and *S. epidermidis* since these pathogens are currently found in infections of patients with BKPro (Eifrig et al., 2003). It was crucial to assess the bactericidal efficacy of the SB_Ag samples during the initial hours to prevent biofilm formation (Thi et al., 2020). The possible bacterial damage was assessed through live/dead staining (Figure 8 **).**

In the live/dead assays, the SB samples showed mostly green-stained bacteria, whereas there were many red-stained bacteria in the SB_Ag samples. The higher penetration of the PI staining is an indicative of bacterial wall damage (Figures 8A, B). The SB_Ag samples exhibited a bacterial reduction of 79.75% for *S. epidermidis* and 87.33% for *P. aeruginosa*. Additionally, higher values of bacterial death were detected in the SB_Ag samples (Figure 8C).

**FIGURE 9:**
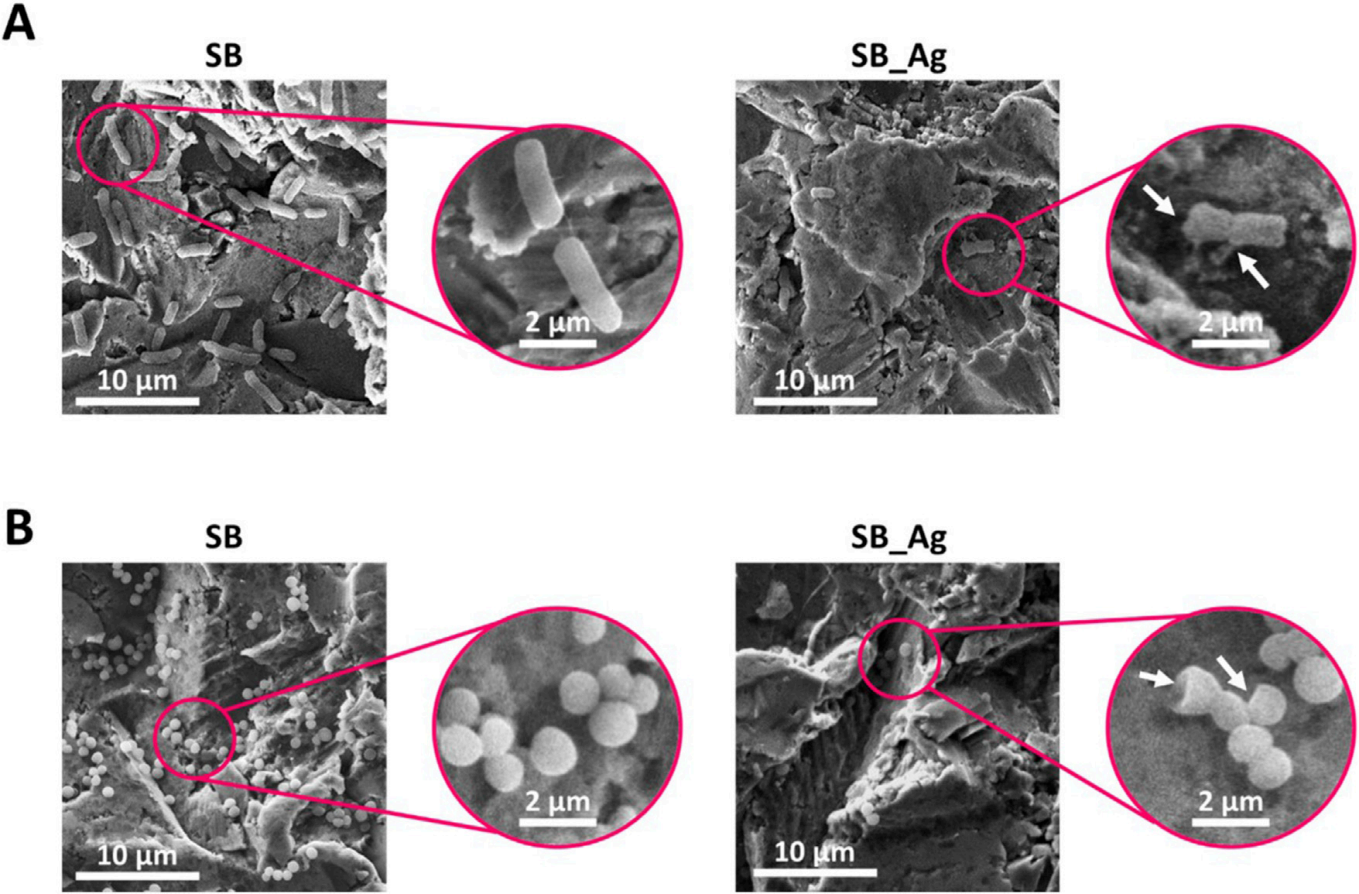
**(A)** Scanning electron microscope (SEM) images of 4 h adhesion of Pseudomonas aeruginosa. **(B)** SEM images of 4 h adhesion of Staphylococcus epidermidis. SB: sandblasted Ti and SB_Ag: sandblasted Ti with silver particles. (scale bars = 10 µm and 2 µm). Arrows indicate bacteria with damaged membranes.

After the confirmation of the ability of the Ag particles to generate damage in both bacterial strains, the samples were also observed through SEM to check possible morphological defects (Figure 9).

In the SEM micrographs, the decrease in bacterial adhesion was confirmed in the SB_Ag samples for both bacterial strains. When observing the samples at higher magnifications, the bacteria in SB samples shown clear definition of the wall, whereas bacteria in the SB_Ag samples clearly showed membrane damage and shrinkage in both studied bacterial strains (Figure 9).

## 4 Discussion

The electrochemical anodization previously reported has been adapted using constant pulse electrodeposition to homogeneously deposit Ag particles on rough Ti surfaces (Godoy-Gallardo et al., 2015a). The proposed treatment was successful, and the presence of Ag deposits firmly attached was corroborated directly by SEM, XPS, and ICP-MS, and indirectly by the observed changes in the wettability of the treated samples.

SEM micrographs confirm the homogeneous adherence and distribution of silver deposits with a globular morphology on the Ti surface, covering the entire surface of the samples. To better visualize these silver particles, images were taken with backscattered electrons. In this way, since silver has a higher atomic number, the Ag deposits appeared brighter in the image. It was verified that Ag precipitates remained attached to Ti even after sonication for 45 min (Figure 2A). This fact is very relevant because the effect of metal ions and wear debris released from metallic implanted medical devices is a concern in the long-term systemic health (Matusiewicz and MRichter, 2021).

Surface roughness values were comparable between samples treated with the electrodeposition technique, SB_Ag (silver-anodized samples) and SB (control samples) conditions (Figure 2B). The values for the SB condition were resembled those obtained in previous studies (Gómez et al., 2023), while the values for SB_Ag samples were similar although with higher deviation. Studies using a similar electrochemical anodizing process on smooth titanium samples showed that roughness (Ra) increase associated with silver deposits ranged between 50–60 nm (Godoy-Gallardo et al., 2015a). Hence, the larger deviation in the results is normal as the silver particles generated nanotexture. Increased variability in roughness due to the presence of silver particles has been previously reported (Lampé et al., 2019).

Regarding wettability, the values obtained for SB were similar to those obtained in previous studies (Gómez et al., 2023), while the values for SB_Ag were higher (Figure 2B). These results are consistent with the XPS findings, where the water signal decreased after the electrodeposition treatment, resulting in an increased hydrophobic character of the SB_Ag samples (Figure 4B). These results are consistent with previous publications, which indicate that an increase in surface discontinuities due to larger convert the fluorescence Ag particles leads to a higher water contact angle (Hajdu et al., 2020). Indirectly, the decreased wettability observed in SB_Ag samples compared to SB samples indicates successful silver deposition.

Quantitative elemental composition of the X-ray photoelectron spectroscopy spectra certifies the successful immobilization of silver on the rough titanium surface. In silver-anodized samples, Ag3d signals were detected in addition to a decrease in percentage of Ti2p and O1s signals. Also, a weak sulphur signal (S2p) is observed, probably due to remains or impurities from the silver complex used with this electrochemical technique (Godoy-Gallardo et al., 2015a). Deconvolution of the Ag 3d high-resolution spectra showed peaks corresponding to silver oxides (Ag_2_O: 367.1 eV, AgO: 366.2 eV) and metallic silver (Ag: 368.4 eV). Notably, the binding energies and Auger parameter values are quite similar for AgO_2_ and AgO, complicating the distinction between the two species. It was observed that silver was mainly in oxide form (84.8%).

Unlike metallic silver, silver oxide exhibits slight solubility in water, primarily due to the formation of ions such as Ag(OH)^2-^ and other related hydrolysis products, which generate positively charged silver ions (Ag^+^). This is important since Ag^+^ ions are renowned antimicrobial agents, effectively targeting bacteria, fungi, and certain viruses.

Silver ions exert their antimicrobial effects through various mechanisms, including the inhibition of bacterial respiration, disruption of bacterial cell membranes, and interference with cell division by binding to DNA (Anees Ahmad et al., 2020; Mikhailova, 2020).

In conducting the cytotoxicity assay, cell viability values were above 80% in all dilutions of the SB_Ag sample extracts. These findings are highly favorable, as the ISO 10993–5 standard stipulates that cell viability must be greater than 80% to consider a material non-cytotoxic (International Organization for Standardization, 2009). Consequently, these results confirm the short-term non-cytotoxicity of the SB_Ag samples (Figure 7A). The initial decrease in cell adhesion in SB_Ag samples was not statistically significant, and there were no significant changes in the shape descriptors (Figure 6). Additionally, the silver deposits showed no effect on the proliferation of HCKs. Therefore, the *in vitro* cell tests carried out indicate the absence of harmful effects on HCKs in the short and long term due to the presence of the silver deposits. In the literature, the HCKs cell line has been utilized for various purposes, including assessing keratocyte-specific toxicity, evaluating the toxicity of fluoroquinolones, and studying the effects of cosmetic and pharmaceutical compounds (Zorn-Kruppa et al., 2004; Bezwada et al., 2008; Shalam et al., 2009). Additionally, HCKs have been employed as building blocks in the development of 3D human corneal models (Paterniti et al., 2024). However, prior to this study, HCKs have not been utilized to test the toxicity of Ag. The effects of Ag have been tested with human corneal epithelial cells or rabbit keratocytes yet not with HCKs (Kim et al., 2021). Despite the low levels of Ag ion release detected in this study via ICP-MS, the silver deposits were tested for potential harmful effects on HCK cells. After confirming their cytocompatibility, the question of whether the silver-treated surfaces exhibited antibacterial properties arose.

This study aimed to test the antibacterial properties of Ag-treated surfaces on bacterial strains commonly associated with KPro infections. *Pseudomonas aeruginosa* is a well-established causative agent of corneal infections, known for its ability to form biofilms and develop antibiotic resistance (Chan and Holland, 2012; Robert et al., 2012; Wagoner et al., 2016b; Hilliam et al., 2020; Urwin et al., 2020). Keratitis caused by *P. aeruginosa* is particularly concerning due to its rapid growth, potentially leading to vision loss after ocular surgery (Hilliam et al., 2020; Paterniti et al., 2024). Despite the significance of studying the microbial flora on the ocular surface of KPro patients, research in this area remains limited. In one study (Eid et al., 2012), *S. epidermidis* and other coagulase negative *Staphylococci* species were identified as common isolates, raising concerns about its increasing prevalence in nosocomial infections, although its role in conjunctivitis or keratitis remains unclear (Flores-Páez et al., 2015). Medical devices are believed to be the primary mode of infection transmission (McCann et al., 2008; Leshem et al., 2011).

Current strategies to combat implant infection include antibiotics and/or steroids; however, the use of these compounds can alter the healthy eye microbiota, leading to resistance of pathogenic strains (Chiang and Chern, 2022). With the aim of preventing infections, it is crucial to develop alternatives that confer antibacterial properties to implants, especially during the first hours post-implantation (Costerton et al., 1999; Høiby et al., 2010). These initial hours are critical for preventing bacterial adhesion or damaging them, thereby preventing infection or the development of biofilms (Campoccia et al., 2013; Thi et al., 2020). Therefore, it was crucial in our study to test the antibacterial effect of Ag-treated surfaces against representative pathogenic bacterial strains in the initial hours.

The Ag-treated samples showed less bacterial adhesion and bacteria with damaged bacterial walls for the two strains studied, indicated by higher PI staining. SEM observation confirmed the presence of bacterial wall damage characterized by shrinkage and wrinkles in both strains. These results are highly promising as they underscore the effectiveness of Ag particles, particularly in the early stages of infection crucial for preventing more complex infections. These findings are consistent with similar work that has reported a decrease in adhesion and growth of several bacterial strains of oral microbiota (Godoy-Gallardo et al., 2015a). Despite the low values Ag^+^ ions detected (40–85 ppb) via ICP-MS, a bactericidal mechanism was evident. There is no consensus on the amount of silver ions required for antimicrobial activity. In one study, the minimum inhibitory concentration (MIC) of Ag^+^ against *P. aeruginosa* was reported to be around 25 ppm (Sharma et al., 2015), while another study reported a MIC of 1 ppm, demonstrating a strong antibacterial effect on both *P. aeruginosa* and *S. epidermidis* (Li et al., 2017). Additionally, S. Rtimi et al. (2013) demonstrated that a silver release of less than 5 ppb/cm^2^ already inhibits bacterial growth (Rtimi et al., 2013). Considering the size of the samples used in this study, the values of Ag^+^ release obtained were 51–108 ppb/cm^2^, higher than those reported by S. Rtimi.

Silver ions exert multiple mechanisms of action, including the disruption of bacterial membranes, intracellular proteins, and DNA, as well as the increase in reactive oxygen species (ROS) levels (Le Ouay and Stellacci, 2015). Among these mechanisms, the interaction of Ag^+^ with reductase enzymes, such as glutathione peroxidases, alters cellular processes to regulate ROS concentrations, ultimately leading to oxidative stress which in turn could result in cellular death (Piao et al., 2011). The generation of ROS and subsequent increase in oxidative stress are widely accepted as the potential mechanisms underlying the toxicity of Ag (Barcińska et al., 2018). Conversely, membrane destabilization and rupture, driven by elevated ROS levels have been reported (Barcińska et al., 2018; Zhang et al., 2019). However, the formation of ROS levels even at low concentrations of Ag has been reported (Park et al., 2009). It was hypothesized that, in our scenario, the bactericidal effect of the Ag-treated surfaces operates through enhanced oxidative damage mediated by ROS. Consequently, even at low concentrations of Ag^+^, mechanisms inducing an increase in ROS concentration remain active, capable of harming bacteria.

Silver has long been valued for its potent antimicrobial properties, which are beneficial in various medical and industrial applications (Kim et al., 2007; Kanmani and Rhim, 2014). However, recent studies have highlighted concerns regarding the potential systemic toxicity of silver compounds, leading to increased regulatory scrutiny and calls for a comprehensive assessment of their benefits and risks (Hadrup and Lam, 2014). The European Union has responded by implementing stringent regulations governing the use of silver in consumer products, cosmetics, and medical devices, with the aim of safeguarding public health and the environment (SCCS, 2024). Our study contributes to this ongoing discussion by demonstrating that silver agglomerates, distinguished by their nanometric size and complexed form rather than nanoparticles, when deposited on titanium (Ti) surfaces, exhibit minimal release of silver ions (Ag^+^). This unique characteristic holds promise for mitigating issues commonly associated with silver nanoparticles (AgNPs), such as cytotoxicity and antibiotic resistance (Foldbjerg et al., 2011; Chernousova and Epple, 2013; Sahu et al., 2014). Despite the potential toxicity of silver ions (Ag^+^), our research reveals that the obtained silver deposits showed reduced release of Ag^+^, suggesting a potential reduction in associated risks (Lok et al., 2006; Manke et al., 2013). In this sense, we have demonstrated that very low silver concentrations provide sufficient antibacterial capacity against two representative bacterial strains in ophthalmic infections. The safety of the silver-obtained surfaces was evaluated through ISO 10993 standard for implantable biomedical devices and no adverse effects were found. Nevertheless, it is crucial to underscore the necessity for further research employing more complex models to comprehensively evaluate the long-term impact of these coatings.

Despite the promising results, this study has limitations that should be considered. The electrodeposition technique presents certain disadvantages, such as replicability issues and non-uniformity of the deposited particles, as reported for this type of technique (Costa et al., 2022). These limitations are significant for the subsequent application of the developed electrodeposition technique in more complex models. Additionally, future studies should include *in vivo* experiments to validate these findings in a clinical setting and explore the long-term efficacy and safety of Ag-treated surfaces.

This study offers detailed physicochemical characterization to elucidate the changes in surface behavior induced by the Ag deposits. Extensive biological characterization has also been carried out to test the safety of the Ag particles in the short and long term, using *in vitro* tests with corneal keratocytes. The results obtained provide valuable information on the effect of Ag on corneal keratocytes. The antibacterial efficacy of the Ag deposits has been corroborated on both gram-positive and gram-negative strains. Additionally, this study outlines constant pulse electrodeposition as an effective technique to modify the Ti backplate of the BKPro with Ag to prevent infections that compromise the implant retention avoiding antibiotic resistance. Overall, this study opens the door to further study the effect of Ag particles on corneal tissue.

## 5 Conclusion

Constant pulse electrodeposition was utilized to effectively deposit firmly attached Ag particles on rough Ti surfaces. The Ti surfaces with Ag particles underwent physicochemical and biological characterization through *in vitro* assays. The Ag-treated surfaces presented silver deposits of Ag mainly in oxide state. The absence of cytotoxic and harmful effects on the adhesion and proliferation of human corneal keratocytes (HCKs) was confirmed. The silver-treated surfaces exhibited a significant antibacterial effect against *P. aeruginosa* and *S. epidermidis*, resulting in bacterial damage and evident perturbations in the bacterial cell wall despite the low release of Ag^+^ ions. The bactericidal mechanism of the silver deposits is likely attributed to an increase in the production of reactive oxygen species (ROS). Further investigations are planned to evaluate the antibacterial effect of the Ag-treated surfaces in a realistic *in vivo* model. Importantly, our study demonstrates that the developed technology yields rough Ti surfaces with firmly anchored Ag particles, antibacterial properties against *P. aeruginosa* and *S. epidermidis*, and good compatibility with corneal tissue. This finding underscores the potential of our approach as a safe and promising alternative to antibiotics to prevent early infections of the Ti backplate of the BKPro. The results obtained in this study, along with the proposed future studies, will contribute to the development of keratoprostheses (KPros) with a lower infection rate.

## Data Availability

The original contributions presented in the study are included in the article/supplementary material, further inquiries can be directed to the corresponding author.
